# Hearing Sensitivity to Gliding Rippled Spectra in Hearing-Impaired Listeners

**DOI:** 10.3390/audiolres14060078

**Published:** 2024-10-24

**Authors:** Dmitry Nechaev, Olga Milekhina, Marina Tomozova, Alexander Supin

**Affiliations:** A.N. Severtsov Institute of Ecology and Evolution, 119071 Moscow, Russia; o-milekhina@mail.ru (O.M.); m.tomozova86@mail.ru (M.T.); alex_supin@mail.ru (A.S.)

**Keywords:** hearing, hearing loss, rippled spectra, spectral–temporal resolution

## Abstract

Objectives: Sensitivity to the gliding of ripples in rippled-spectrum signals was measured in both normal-hearing and hearing-impaired listeners. Methods: The test signal was a 2 oct wide rippled noise centered at 2 kHz, with the ripples gliding downward along the frequency scale. Both the gliding velocity and ripple density were frequency-proportional across the signal band. Ripple density was specified in ripples/oct and velocity was specified in oct/s. The listener’s task was to discriminate between the signal with gliding ripples and the non-rippled signal. Results: In all listener groups, increasing the ripple density decreased the maximal velocity of detectable ripple gliding. The velocity limit of ripple gliding decreased with hearing loss. Conclusions: The results can be explained by deteriorated temporal resolution in hearing-impaired listeners.

## 1. Introduction

Hearing impairment deteriorates the ability to distinguish suprathreshold sounds with complex spectra, which are the spectra characteristic of speech sounds.

The deterioration of hearing sensitivity and sound discrimination largely appears due to dysfunction in the inner ear; in particular, because of low gain in the active cochlear mechanism based on the activity of the outer hair cells. This low gain in the active mechanism results in lower-quality cochlear-frequency-tuned filters and, hence, poor frequency tuning, less pronounced lateral suppression, and compression [1].

Apart from that, deterioration in hearing selectivity and speech recognition in hearing-impaired listeners may be the result of a lessened ability to analyze the temporal fine structure of sounds and an inability to process rapid sound variations [2,3].

One more mechanism of hearing impairment that remains under question is discriminating signal envelope modulation. Some studies have shown that age and hearing loss do not affect amplitude modulation (AM) frequency selectivity [4,5]. Other studies have demonstrated that age, independent of hearing loss, negatively impacts AM frequency selectivity, while hearing loss improves AM detection and AM selectivity, likely due to loss of the compression mechanism [6,7].

The ability to discriminate complex spectral–temporal patterns may be assessed using test signals with rippled spectra. These spectra feature alternating spectral amplitude maxima and minima.

Signals with a linear distribution of ripples on the frequency axis are used to study the effect of repetition pitch [8], where the pitch perception depends on the interval between the ripples. A deteriorated ability to perceive the pitch of iterated rippled noises has been demonstrated in listeners with hearing impairment [9].

Signals with a logarithmic ripple distribution are used to study spectral and spectral–temporal discrimination. The spectral resolution of hearing (the frequency-resolving power) is determined by the maximum resolvable ripple density [10]. Additionally, the resolvable ripple depth may be considered a measure of spectral modulation resolution (spectral contrast sensitivity) [11,12].

Various discrimination tasks can be used in conjunction with different versions of rippled-spectrum tests: (i) discrimination between a rippled test signal with multiple ripple reversals and a flat spectrum (the spectral ripple discrimination test) [13,14,15]; (ii) discrimination between a flat spectrum and a rippled spectrum with varying modulation depths (the spectral ripple detection test) [15,16]; and (iii) discrimination between a ripple spectrum with a gliding ripple phase and a constant rippled or flat spectrum (the spectral-temporally modulated ripple test) [17,18,19].

A test using ripples gliding upward or downward along the frequency scale has been suggested as a version of rippled-spectrum signals varying in time [20,21].

For all these tests, results worsen in listeners with hearing impairment. Discrimination, assessed by ripple density, reduces by approximately two-fold [22,23]. This reduction may be due to wider passbands in frequency-tuned filters, resulting in smeared excitation patterns and an inability to reproduce high ripple densities.

In spectral–temporal tests, signal discrimination depends on ripple density (measured in ripples/oct) and the temporal modulation rate (measured in Hz). These signals contain elements of spectral and temporal modulation, as well as temporal fine structure information [18,24,25,26]. Thus, this signal type can assess how the auditory system analyzes temporal patterns in sound signals. Notably, a rippled signal can be characterized by a limited number of parameters, including, specifically, ripple density, ripple depth, ripple position on the frequency scale, direction, and the velocity of ripple gliding.

With the use of rippled-spectrum test signals, thresholds for spectral ripple depth have been assessed for both normal-hearing and hearing-impaired listeners. For hearing-impaired listeners, an increased ripple depth threshold was demonstrated for certain combinations of ripple density and ripple gliding velocity. At low temporal modulation rates, increased thresholds result from reduced temporal resolution [24,25].

In both normal-hearing listeners and cochlear implant users, increasing the ripple density results in higher ripple depth thresholds at a temporal modulation rate of 5 Hz [27]. Therefore, in recent investigations, gliding ripple signals have been exploited to measure spectral resolution. However, combined spectral–temporal resolution has not been measured in those studies as gliding velocity was not varied or varied within a small range, and its influence on signal discrimination has not been investigated. In one study [26], the maximal temporal modulation rate decreased from 388 Hz at a ripple density of 1 ripple/oct to 79 Hz at 7 ripples/oct.

Considering the ripple pattern resolution’s dependence on both ripple density and gliding velocity, the present study aimed to investigate the resolution of ripple patterns as a function of both ripple density and gliding velocity in listeners with normal hearing and hearing impairment.

## 2. Materials and Methods

### 2.1. Listeners and Experimental Conditions

Twenty-two listeners with different hearing sensitivity participated in this study. The ages of the listeners ranged from 19 to 85 years. To categorize the degree of hearing loss, the Global Burden of Disease Expert Group on Hearing Loss scale was used [28]. In this classification, the average tonal thresholds at four frequencies are taken into account: 0.5, 1, 2, and 4 kHz. According to this scale, six listeners (ages 19 to 38) had excellent hearing (10 to 4.9 dB hearing level), six listeners (ages 23 to 70) had good hearing (5 to 19.9 dB), six listeners (ages 52 to 75) had mild hearing loss (20 to 34.9 dB), and five listeners (ages 67 to 85) had moderate hearing loss (35 to 49.9 dB).

During the experiments, the listeners were in a soundproof cabin (MINI 350, IAC, Germany, Moenchenglabach) that attenuated external sounds by no less than 40 dB.

### 2.2. Tonal Audiometry

**Figure 1:**
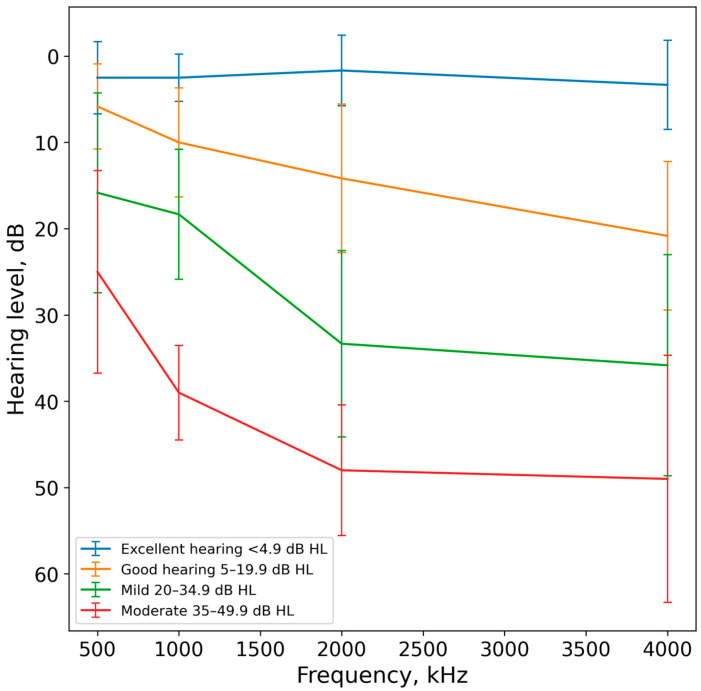
The mean audiograms for four groups of listeners with different hearing sensitivity. The error bars represent standard deviations.

**Figure 2:**
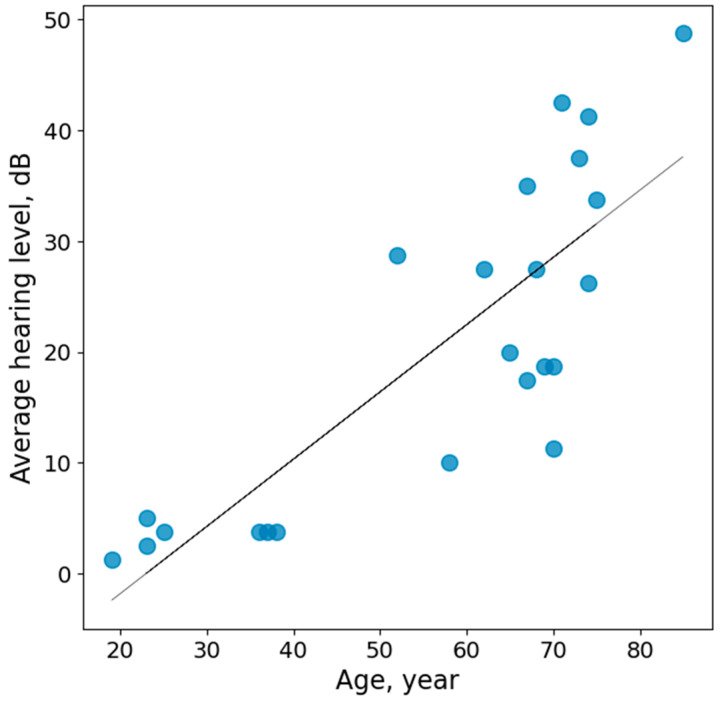
Average hearing level as a function of listeners age. The points represent individual data.

Tonal thresholds were measured within a frequency range of 0.25 to 8 kHz, using an AA-02 audiometer (Biomedilen, St. Petersburg, Russia) (Figure 1 and Figure 2).

### 2.3. Signals

The test signals were band-limited rippled noise. The envelope of the spectrum was a two-octave-wide cycle of a log frequency cosine function. The cosine envelope was used to avoid the effects of spectrum edges, which can influence the resolution of a rippled-spectrum noise burst. The spectrum’s central frequency was 2 kHz and covered a frequency band of 1 to 4 kHz. Within the envelope, the spectrum had spectral ripples. The ripples were defined by a log frequency cosine function. A common metric for this type of ripple pattern is the number of ripples per octave (ripples/oct). The ripple density values were 1, 1.5, 2, 3, 5, 7, and 10 ripples/oct.

During the test signal, ripples glided within the band with a velocity that was also frequency-proportional; that is, it was constant across the signal band when specified in oct/s. The velocity values included but were not limited to 1, 1.5, 2, 3, 5, 7, 10, 15, and 20 oct/s (a quasilogarithmic scale). Only downward-gliding ripples were used.

The reference signal was a non-rippled noise burst. Its spectrum reproduced the envelope of the test signal. The root-mean-square levels of the test and reference signals were equalized.

Signal level was selected depending on the listener’s tone detection threshold at a 2 kHz frequency using the following formula:

L=70+0.5∗T

where *L* (dB sound pressure level, SPL) is the level, and *T* (dB SPL) is the tone detection threshold at the central frequency of the spectrum signal. Using this formula, the minimum signal level used for listeners without hearing loss (T = 0 dB) was found to be 70 dB SPL, and the maximum signal level for listeners with hearing loss (T = 60 dB) was 100 SPL. This formula satisfactorily approximated the signal levels selected by the subjects as comfortable for discriminating the test and reference signals without discomfort due to excessive volume.

### 2.4. Signals Generation

**Figure 3:**
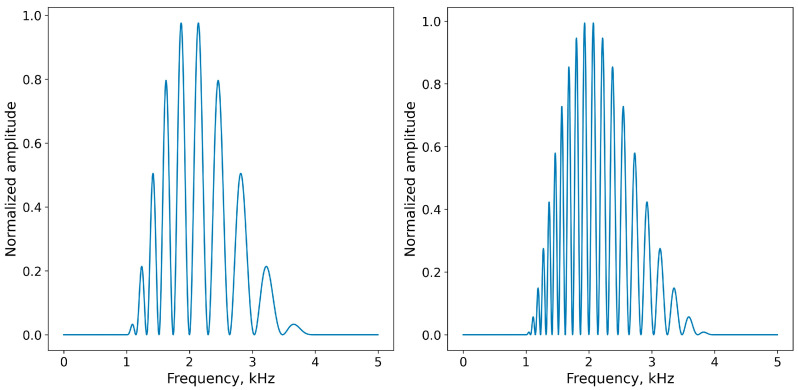
Example of a ripple spectrum with different densities (left—5 ripples/oct; right—10 ripples/oct); central frequency of 2 kHz and frequency bandwidth of 2 oct.

The signal generation method was similar to that used in [26]. The signals were digitally generated at a sampling rate of 32 kHz. To generate a test signal, a wide-band signal was digitally filtered by a filter, as presented in Figure 3. Signals with equal noise parameters were not exact copies of each other, but differed by fluctuations intrinsic to noise.

The filter form was changed in such a way that ripple positions shifted by

(1) δf=v/r,

where δf (oct) is the sample-by-sample shift in the ripple position, v (oct/s) is the gliding velocity, and r is the sampling rate.

Each signal lasted 2 s (64,000 samples). The result of this procedure was a 2 s long ripple-gliding noise burst.

**Figure 4:**
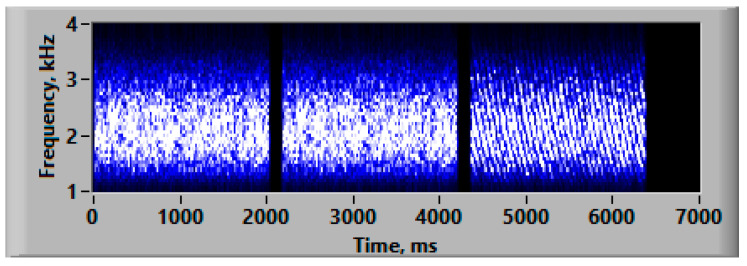
Spectrograms of the two reference signals (first and second) with a non-rippled structure and the test signal (third) with a density of 3 ripples/oct and a gliding velocity of 5 oct/s.

The same method was used to generate a reference signal, except that the filter did not have a rippled structure. The result of this procedure involved two non-rippled noise bursts with 2 s long durations. Examples of signal spectrograms are presented in Figure 4.

### 2.5. Experimental Procedure

Measurements of the ripple gliding resolution were performed using a three-alternating forced-choice procedure with adaptive variation in the ripple gliding velocity. In each trial, the listeners heard a sequence of three signals: one test and two reference signals. Each signal lasted 2000 ms, with 100 ms pauses between them. The order of the signal presentation varied randomly, trial by trial. The listener’s task was to report which of the three signals differed from the other two. The listeners were not instructed to pay attention to any specific cue that might distinguish the test signal from the reference signals. To better understand the task, listeners were given feedback on whether they accurately or incorrectly identified the test signal.

The ripple gliding velocity was varied adaptively using a two-up, one-down paradigm. After two correct answers, the velocity in the next trial increased by one step; after a mistake, the velocity decreased by one step. This adaptive procedure tracked the velocity around a value that resulted in a probability of correct detections of 0.71 [29]. Each measurement procedure continued until 10 reversal points (transitions between velocity increase and decrease) were obtained. The geometric mean of these 10 reversal points was taken as the resolution estimate.

Geometric rather than arithmetic means were used because ripple density was varied in steps closer to a logarithmic scale than a linear scale.

For each combination of ripple density and gliding velocity combination, measurements were repeated three times for each listener. The arithmetic means of the three results were taken as the final resolution estimate for each listener.

### 2.6. Instrumentation

The signals were digitally synthesized on a standard personal computer using a custom-made program designed with LabVIEW (National Instruments, Austin, TX, USA). The digitally generated signals were converted from digital to analog using a 16-bit converter in a data acquisition board (NI USB-6251, National Instruments). The analog signals were played diotically through headphones (Sennheiser HD 650, Sennheiser, Wedemark, Germany). All statistical analyses were performed using Python 3.9.18.

## 3. Results

### 3.1. Origin of Hearing Impairment

For the listeners in the present study, hearing impairment was age-dependent, as confirmed by both the correlation between age and hearing impairment and the slopes of the regression lines (*p* < 0.001, r = 0.8) (Figure 2). The absence of any history of hearing-related traumas among the listeners further supports the age-dependent origin of their hearing loss.

### 3.2. Relationship between Ripple Density Resolution and Gliding Ripple Velocity

**Figure 5:**
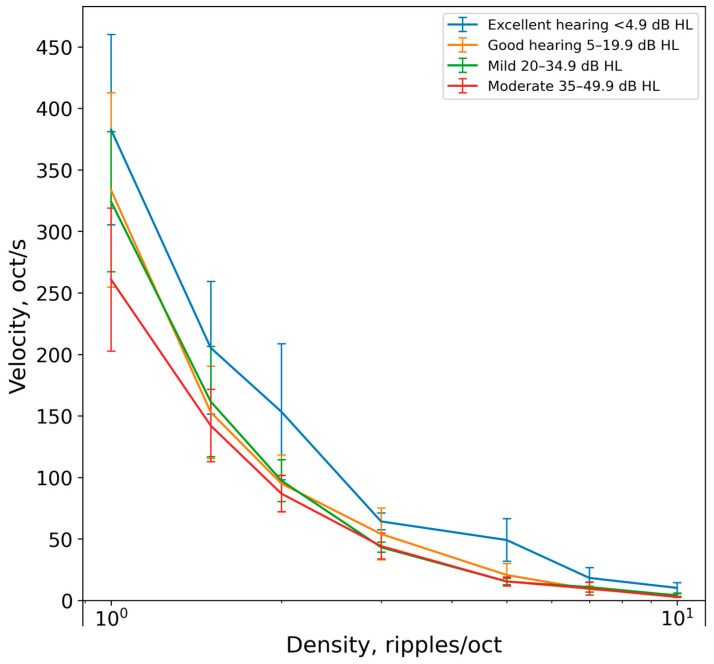
Gliding velocity limits as a function of ripple density. Data are shown for four listener groups with different hearing sensitivity. Error bars represent standard deviations.

The resolution limit of the gliding ripples depended on ripple density: the higher the ripple density, the lower the velocity limit. This relationship was characteristic of all listener groups (Figure 5).

At the lowest ripple density of 1 ripple/oct, the mean velocity limit ranged from 382 (the group with excellent hearing) to 260 oct/s (the group with moderate hearing loss) (or from 382 to 260 ripples/s or Hz). At the highest ripple density of 10 ripples/oct, the mean velocity limit ranged from 10 (the group with excellent hearing) to 2 oct/s (the group with moderate hearing loss) (or from 1 to 0.2 Hz).

The Kruskal–Wallis test revealed statistically significant differences in the ripple gliding thresholds for densities of 2 (*p* = 0.009), 3 (*p* = 0.03), 5 (*p* = 0.03), and 10 ripples/oct (*p* = 0.003) between groups of listeners. For a density of 1 ripple/oct, the *p*-value was 0.058.

Pairwise comparisons were performed using Dunn’s test with a Bonferroni correction for multiple comparisons. The differences between the groups were as follows:

- At a ripple density of 1 ripple/oct, the difference between the Excellent and Moderate groups resulted in *p* = 0.038;
- At a density of 2 ripples/oct, the differences were as follows: Excellent vs. Moderate (*p* = 0.014) and Excellent vs. Good (*p* = 0.034);
- At a density of 3 ripples/oct, the difference between the Excellent and Mild groups resulted in *p* = 0.056;
- At a density of 5 ripples/oct, the differences were as follows: Excellent vs. Mild (*p* = 0.005) and Excellent vs. Moderate (*p* = 0.016);
- At a ripple density of 10 ripples/oct, the difference between the Excellent and Moderate groups resulted in *p* = 0.003.

Thus, the statistical test confirmed the significance of differences only between the extreme listener groups (the group with excellent hearing and the group with moderate hearing loss).

### 3.3. Relationship Between Ripple Density Resolution and Tone Detection Thresholds

**Figure 6:**
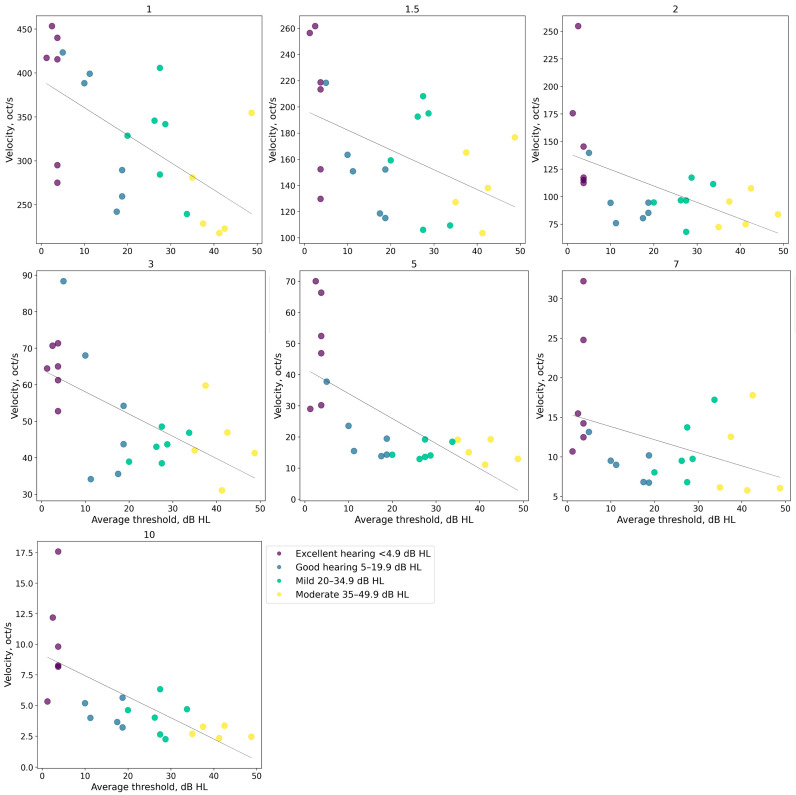
Gliding velocity limits as a function of average thresholds for ten ripple densities. Each panel represents results for one density. Different colors represent different groups of listeners, each point represents individual data, and the straight lines are regression lines.

To further analyze the interrelation between the ripple gliding limits and hearing loss, both regressions and correlations were computed between these limits and the average tonal thresholds (Figure 6).

The results of the correlation and regression analyses are presented in Table 1.

For all ripple densities except 7 ripples/oct, there were statistically significant negative trends and statistically significant correlations ranging from −0.5 to −0.69.

The analysis indicated that, independent of ripple density, increased tonal thresholds were associated with decreased ripple gliding limits.

## 4. Discussion

In the present study, ripple density was specified in ripples/oct, i.e., the ripple width was frequency-proportional. The ripple gliding specified in oct/s was also frequency-proportional. Therefore, the rate of fluctuations in the spectral components (expressed in Hz) that appear while ripples glide was equal across the frequency scale.

Wide-band test signals with equally spaced ripples produced a sensation of pitch that was equal to the ripple frequency spacing (the repetition pitch) [8,30,31]. The repetition pitch was the strongest for ripple temporal fluctuations of approximately 500 Hz. The fluctuation rates below 50 and above 2000 Hz produced no repetition pitch. The maximal sensation of a repetition pitch of 500 Hz was a little higher than the limits of 260 to 382 Hz found in the present study, whereas the limit of 2000 Hz was markedly higher. However, notably, the test stimuli in the present study were band-limited, unlike the wide-band stimuli used in studies of repetition pitch. This difference might influence the results.

The results of the present study for normal-hearing listeners were close to those obtained earlier using similar band-limited test signals [26].

Thus, both previous investigations and the present study show that, in normal listeners, the ability of the auditory system to resolve ripple fluctuations is in the range of several hundred Hz. This limit is substantially higher than the limit of perception of tonal fluctuation that was assessed as 50–70 Hz in [32,33]. A fluctuating tone addresses a single frequency, whereas, in gliding ripples, fluctuations appear within a certain frequency band. Alternatively, the higher ripple fluctuation limit might be explained by the temporal-processing mechanism. The role of temporal processing in analyzing rippled-spectrum signals has been demonstrated previously [8].

Age-dependent hearing impairment deteriorates the ability to discriminate the velocity of spectral ripple gliding, impacting both high-density and low-density spectra. A possible mechanism of this lowering may be decreased hearing filter qualities. Because of this decrease, the threshold ripple depth in the excitation pattern is achieved at lower ripple densities, which leads to lower ripple gliding velocities.

Another reason for the decreased resolution of gliding ripples may be deterioration in the temporal processing mechanism. At low temporal resolution, quick fluctuations during high-velocity ripple gliding may not be resolved.

In [20], at a fluctuation frequency of 5 Hz, the highest ripple density that allowed for the detection of ripple gliding was 5–6 ripples/oct in normal-hearing listeners and 1–2 ripples/oct in hearing-impaired listeners; conversely, in [24,25], hearing-impaired listeners discriminated a ripple density of 4 ripples/oct at a fluctuation frequency of 32 Hz. In this study, higher gliding velocities were also observed for both normal-hearing and hearing-impaired listeners. This may be related to the type of reference signal used (rippled or flat). These data were obtained with flat reference signals and might be explained by the temporal processing mechanism. For rippled reference signals that should be distinguished from rippled test signals, the temporal processing mechanism may be less effective because of the similarity between the autocorrelation functions; in this case, the spectral mechanism may be more effective [34].

The authors of [21] hypothesized that discriminating gliding ripples can be explained by amplitude fluctuations with a rate equal to the temporal repetition rate that occurs at the high-frequency edge of the spectrum. To avoid this effect, a cosine form of the ripple spectrum was used in the present study. Additionally, it has been suggested [7] that detecting amplitude modulation does not deteriorate with age-dependent hearing impairment. If so, velocity limits should be independent of or only slightly dependent on listener groups with different hearing sensitivities. This was not the case in the present study.

Notably, owing to the large variability in thresholds within the groups, only a general trend in the spectral–temporal resolution changes that come with age-dependent hearing impairment can be identified from the obtained data.

## 5. Conclusions

This study demonstrated that age-dependent hearing impairment decreases hearing sensitivity (increased threshold) and lowers the resolved gliding ripple velocity. Thus, age-dependent hearing impairment manifests as worse discrimination of complex spectral–temporal patterns in input sounds. This result can be explained either by decreased hearing filter qualities or by deterioration in temporal processing. We believe that a further investigation of a group of age-matched listeners with normal hearing sensitivity would be of interest. In audiology, the results may be used to assess the effectiveness of analyzing the frequency–temporal auditory patterns in age-dependent hearing-impaired listeners.

## Figures and Tables

**Table 1:** Results of the regression and correlation analyses for the dependence of the ripple gliding velocity limit on the threshold of each ripple density.

Density	Slope	Std_Err	*p*_Value	r_Value
1	−3.12	0.91	0.002	−0.6
1.5	−1.51	0.6	0.02	−0.5
2	−1.49	0.5	0.008	−0.54
3	−0.61	0.17	0.002	−0.62
5	−0.8	0.19	0.0003	−0.69
7	−0.17	0.086	0.069	−0.39
10	−0.17	0.04	0.0005	−0.68

## Data Availability

The data that support the findings of this study are available from the corresponding author, DN, upon request.
